# The influence of maternal unhealthy diet on maturation of offspring gut microbiota in rat

**DOI:** 10.1186/s42523-022-00185-w

**Published:** 2022-05-12

**Authors:** Kyoko Hasebe, Michael D. Kendig, Nadeem O. Kaakoush, Aynaz Tajaddini, R. Frederick Westbrook, Margaret J. Morris

**Affiliations:** 1 School of Medical Sciences, Faculty of Medicine and Health, UNSW Sydney, Sydney, NSW 2052 Australia; 2 School of Psychology, UNSW Sydney, Sydney, NSW Australia

**Keywords:** Gut microbiota development, Vertical microbiota transmission, Cafeteria diet, Maternal overnutrition

## Abstract

**Background:**

Despite well-known effects of diet on gut microbiota diversity, relatively little is known about how maternal diet quality shapes the longitudinal maturation of gut microbiota in offspring. To investigate, we fed female rats standard chow (Chow) or a western-style, high-choice cafeteria diet (Caf) prior to and during mating, gestation and lactation. At weaning (3 weeks), male and female offspring were either maintained on their mother’s diet (ChowChow, CafCaf groups) or switched to the other diet (ChowCaf, CafChow). Fecal microbial composition was assessed in dams and longitudinally in offspring at 3, 7 and 14 weeks of age.

**Results:**

The effect of maternal diet on maturation of offspring gut microbiota was assessed by α- and β-diversities, Deseq2/LEfSe, and SourceTracker analyses. Weanling gut microbiota composition was characterised by reduced α- and β-diversity profiles that clustered away from dams and older siblings. After weaning, offspring gut microbiota came to resemble an adult-like gut microbiota, with increased α-diversity and reduced dissimilarity of β-diversity. Similarly, Deseq2/LEfSe analyses found fewer numbers of altered operational taxonomic units (OTUs) between groups from weaning to adulthood. SourceTracker analyses indicated a greater overall contribution of Caf mothers’ microbial community (up to 20%) to that of their offspring than the contribution of Chow mothers (up to 8%). Groups maintained on the maternal diet (ChowChow, CafCaf), versus those switched to the other diet (ChowCaf, CafChow) post-weaning significantly differed from each other at 14 weeks (Permutational Multivariate Analysis of Variance), indicating interactive effects of maternal and post-weaning diet on offspring gut microbiota maturation. Nevertheless, this developmental trajectory was unaffected by sex and appeared consistent between ChowChow, CafCaf, ChowCaf and CafChow groups.

**Conclusions:**

Introducing solid food at weaning triggered the maturation of offspring gut microbiota to an adult-like profile in rats, in line with previous human studies. Postweaning Caf diet exposure had the largest impact on offspring gut microbiota, but this was modulated by maternal diet history. An unhealthy maternal Caf diet did not alter the developmental trajectory of offspring gut microbiota towards an adult-like profile, insofar as it did not prevent the age-associated increase in α-diversity and reduction in β-diversity dissimilarity.

**Supplementary Information:**

The online version contains supplementary material available at 10.1186/s42523-022-00185-w.

## Introduction

The gut microbiota has significant impacts on host growth and development. Evidence shows that microbiota colonization in early life plays a critical role in the establishment and development of gut microbial community in later life, in turn influencing a range of health outcomes [1, 2]. Vertical microbial transmission from mother to offspring has been documented in animals [3, 4] and humans [5–8]. Accumulating evidence implicates maternal gut microbiota as a significant source of offspring gut microbiota colonization. Development of the gut microbiota is shaped by interactions with host genetics, age, diet, and living conditions [9].

Diet is a critical determinant of gut microbiota diversity. An unhealthy maternal diet affects offspring microbial communities, with common species identified in mother and offspring microbial communities [10, 11]. However, it remains to be determined whether the longitudinal maturation of offspring gut microbiota is affected by an unhealthy maternal diet.

Mothers’ habitual dietary patterns considerably shape the dietary patterns of their offspring [12–15]. Given that offspring are likely to consume a habitual dietary pattern similar to that of their mother, it is critical to identify whether the shared microbiota characteristics of mothers and their offspring result from consuming a common diet, or from vertical transmission of gut microbiota in early life. More specifically, it is unknown whether different types of maternal diet can have adverse effects on offspring gut microbiota maturation.

To address this knowledge gap, we used a rat model to test how maternal diet affects the development of the microbiota in offspring, and the extent to which these effects persist when offspring are (a) maintained on the same diet (maternal diet = postnatal diet) or (b) shifted to a different diet (maternal diet ≠ postnatal diet). We assessed the effects of highly processed foods eaten by people by using a validated Cafeteria-style diet (Caf) model [16] on faecal microbial communities in mothers, and their male and female offspring at 3 (weaning), 7 and 14 weeks of age. This design allows us to assess the contributions of species associated with maternal microbiota to offspring microbial community over time. The primary aim of this study was to investigate whether maternal diet affects the development of the microbiota in offspring, and the extent to which these effects interact with offspring diet. Phenotypic changes in offspring including body weight and adiposity were reported in Tajaddini et al. [17]. We found that maternal Caf consumption alters offspring gut microbial communities at weaning; however, after the introduction of solid food, the trajectory of offspring gut microbiota maturation was not affected by maternal Caf diet, and more strongly impacted by offspring diet post-weaning.

## Methods

### Ethics statement

The experimental protocol was approved by the Animal Care and Ethics Committee of the University of New South Wales (Ethics number: 19/74A) in accordance with the guidelines for the use and care of animals for scientific purposes 8^th^ edition (National Health and Medical Research Council, Australia).

### Subjects (and experimental design)

**Fig. 1:**
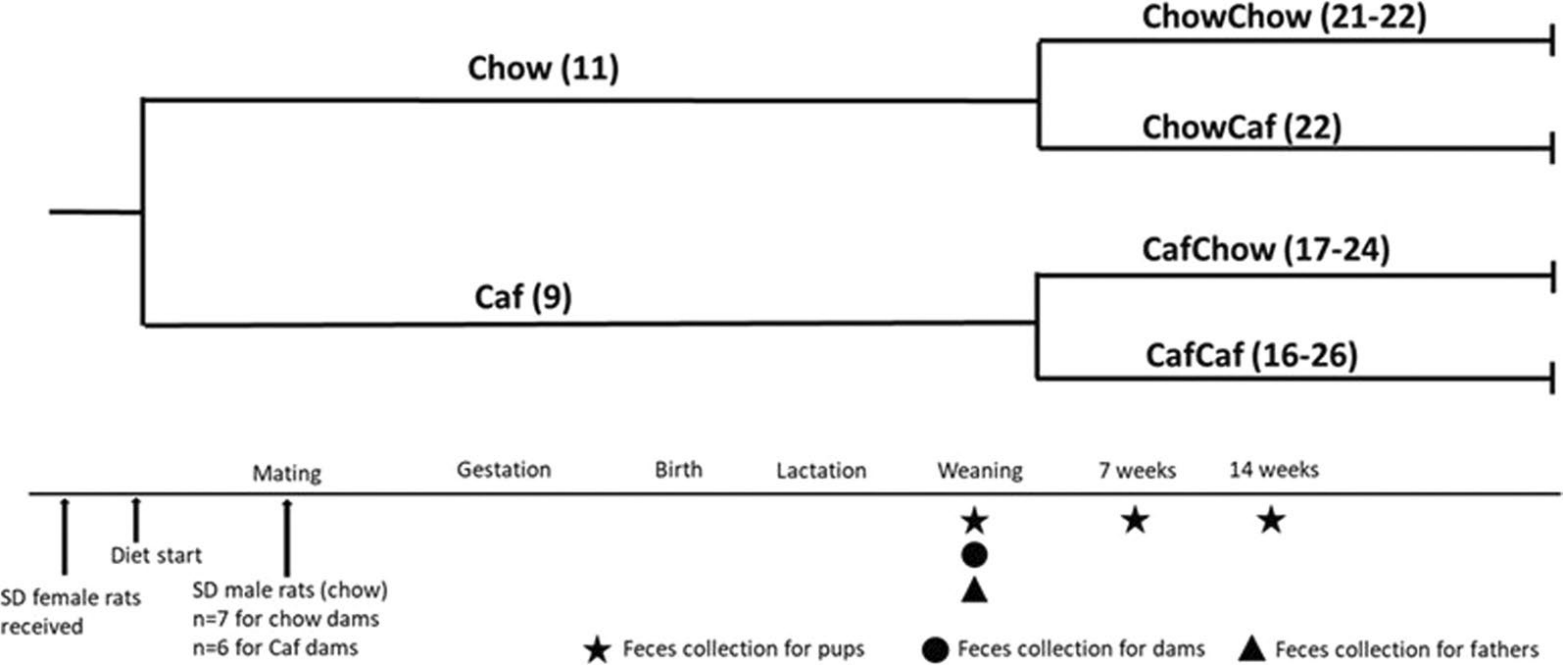
Experimental design. Female rats were fed chow or Caf diet for 6 weeks, then mated with chow fed males. Fecal samples were collected from mothers and pups at weaning (3 weeks of age). Pups were weaned onto chow or Caf diet, and feces were sampled from a subset of offspring at 7 weeks, and from all remaining pups at 14 weeks of age. Experimental groups consisted of Chow Father; Chow Mother; Caf Mother; Chow Weaner; Caf Weaner; ChowChow; ChowCaf; CafChow; CafCaf at both 7 weeks and 14 weeks; ChowCaf 14 weeks; CafChow 14 weeks; and CafCaf 14 weeks

This is a secondary study from a cohort of obese and lean mothers previously described [17]. Young adult female (approximately 7–8 weeks of age; body weight ~ 200 g) and male (approximately 8–9 weeks of age; body weight ~ 300 g) Sprague–Dawley rats (Animal Resource Centre, WA, Australia) were housed at 18–22 °C (12 h light/dark cycle) and maintained ad libitum on water and standard chow (14 kJ/g, 65% carbohydrate, 22% protein and 13% fat; Premium Rat Maintenance diet, Gordon’s Stockfeeds, NSW, Australia). Following acclimatization, female rats were weight-matched, then randomly allocated to either standard chow (Chow) or Caf diet groups. Diets comprised standard chow or chow plus Caf diet consisting of chow and water, 10% sucrose solution and a selection of cakes, biscuits and protein sources (e.g., meat pie and dim sims), that varied daily [16]. Detailed macronutrient and micronutrient components of the Caf diet have been previously described [16]. After 6 weeks of diet, females were mated with chow fed males by co-housing 2–3 females and one male for five days, after which males were removed [17]. Pregnancy was inferred based on weight gain and females were housed individually from approximately gestation day 16. Litters were standardized to six male and six female pups, where possible [17] on postnatal day (PND) 1. Dams and offspring were weighed every three days during lactation. The experimental design is shown in Fig. 1. At weaning (PND20) and at 14 weeks of age, offspring were anaesthetized by i.p. injection of ketamine/xylazine, and decapitated. Retroperitoneal (RP) adipose tissue was collected bilaterally, identified as the triangular pad of fat attached to the lateral abdominal wall; feces were collected at PND20, 14 weeks (at cull) and to provide an intermediate timepoint, an extra collection was taken at 7 weeks in conscious rats.

### Fecal DNA extraction

Fecal DNA extraction was performed using the PowerFecal DNA Isolation Kit (MoBio Laboratories, Carlsbad, CA, USA) according to the manufacturer’s instructions. DNA concentration and quality were measured using a DeNovix DS-11 Spectrophotometer (DeNovis, Inc., Delaware, USA).

### 16S rRNA amplicon sequencing and raw data analysis

Composition of the microbial communities was assessed by Illumina amplicon sequencing (2 × 250 bp MiSeq chemistry, V4 region, 515F-806R primer pair; Ramaciotti Center for Genomics, UNSW Sydney) using a standard protocol [18]. The sequence data were then analyzed using MOTHUR [19], which included removal of ambiguous bases and homopolymers longer than 15 base pairs, alignment with SILVA database, chimera checking with UCHIME, classification against the RDP Ribosomal Database training set (version16_022016), and removal of singletons. Sequences were clustered into operational taxonomic units (OTU) at 97% nucleotide identity to generate an OTU table with the taxonomy and number of sequences per OTU in each sample. Commands were derived from MiSeq SOP [20] and modified as required. Sequence data were subsampled to n = 30,113 total clean reads/sample.

### Data analysis

OTU tables were standardized by dividing feature read counts by total number of reads in each sample. Standardized data were then square root transformed. All statistical analyses examined sex-specific differences in the offspring. α-diversity (species richness, species evenness and Shannon’s diversity index) analyses, Non-metric Multi-dimensional Scaling (NMDS) plots, Permutational Multivariate Analysis of Variance (PERMANOVA) and Permutational Analysis of Multivariate Dispersions (PERMDISP) were generated using a Bray–Curtis resemblance matrix on PRIMER (Primer-e Ltd., Plymouth, United Kingdom) [20]. Linear Discriminant Analysis (LDA) Effect Size (LEfSe) [22] was performed using the Galaxy web application [23]. The R package Phyloseq [24] was used for the negative binomial Wald test in DESeq2 [25]. *P* values were adjusted for multiple testing using Benjamini–Hochberg false discovery rate correction in DESeq2. Differentially abundant OTUs were defined as those that were present in over 50% of rats, and which were significantly different in both LEfSe and DESeq2 analyses. SourceTracker [26] analysis assessed similarities between maternal and offspring microbiota via the Galaxy web application [23, 27].

Statistical analyses were performed with SPSS, including independent samples *t* tests, one way and two-way Analysis of variance (ANOVA). For α-diversity measures (Species richness, Evenness and Shannon index), independent samples *t* tests were used to compare chow and Caf mothers. Two-way ANOVA (maternal diet × sex) was used for comparisons between weanlings. Four-way ANOVA was used to assess differences between the four offspring groups (ChowChow, ChowCaf, CafChow, CafCaf) at 7 and 14 weeks of age (2 (maternal diet) × 2 (postnatal diet) × [2] (time) × 2 (sex)). β-diversity was examined by PERMANOVA, with a single factor of diet for mothers (Chow and Caf), and two factors (diet and sex) for weanlings. For postweaning data, four factors (maternal diet, postnatal diet, age, and sex) were used in analysis of overall offspring microbial composition, followed by PERMDISP analysis. Figures were generated in GraphPad Prism and PRIMER. Results are expressed as mean ± SEM and were considered significant at *p* ≤ 0.05.

## Results

### Influence of maternal and postnatal diets on body weight

Table 1 shows anthropometric data of rats used in this study which was a subset of animals from our previous study [17]. The Caf diet increased maternal consumption of sugar, saturated fat and protein, relative to chow diet [17]. Prior to mating, mothers fed the Caf diet had significantly elevated adiposity but no significant change in fasting blood glucose. When euthanized after lactation, RP fat mass was still elevated in Caf versus control dams, with no difference in body weight or girth [17].

**Table 1:** Anthropometric data of animals used in this study

	Group (n)	Body weight (g) Diet day 30	Body weight (g) At mating	Body weight (g) At weaning	RP fat (g) at kill (1d post-weaning)
Mothers	Chow (11)	278.7 ± 3.5	282.7 ± 8.3	393.4 ± 7.8	2.93 ± 0.24
	Caf (9)	336.2 ± 7.2****	347.2 ± 11.3***	393.8 ± 8.7	4.38 ± 0.56*
Weanlings	Group (N; M/F)	Body Weight (g) (male; female)		RP fat (g) (male; female)*^#^	
	Chow mother (21;10/11)	45.91 ± 2.33	43.95 ± 1.97	0.07 ± 0.01	0.05 ± 0.01
	Caf mother (16;9/7)	42.14 ± 1.28	39.83 ± 1.25	0.17 ± 0.02	0.09 ± 0.01
7 weeks	Group (N; M/F)	Body Weight (g) (male; female)^##ϮϮ^		RP fat (g) (male; female)	
	ChowChow (21;11/10)	370.4 ± 7.8	226.7 ± 5.5	n/a	n/a
	ChowCaf (22;12/10)	411.5 ± 12.3	274.8 ± 8.0	n/a	n/a
	CafChow (17;9/8)	370.7 ± 6.2	227.1 ± 4.8	n/a	n/a
	CafCaf (16;9/7)	433.0 ± 9.8	276.0 ± 5.8	n/a	n/a
14 weeks	Group (N; M/F)	Body weight (g) (male; female)*^##ϮϮ^		RP fat (g) (male; female) ^##ϮϮ^	
	ChowChow (22;11/11)	581.2 ± 12.8	309.5 ± 9.6	4.31 ± 0.40	2.64 ± 0.58
	ChowCaf (22;11/11)	724.1 ± 24.4	435.8 ± 32.2	12.96 ± 0.84	7.20 ± 0.77
	CafChow (24;13/11)	619.0 ± 11.3	312.9 ± 10.1	5.52 ± 0.55	2.62 ± 0.37
	CafCaf (26;13/13)	794.1 ± 17.6	433.7 ± 16.6	15.19 ± 0.93	6.14 ± 0.37

Data are displayed as mean ± SEM

Mothers: Body weight across time was analysed by mixed repeated measures ANOVA with Sidak’s multiple comparisons test and Retroperitoneal (RP) fat was analysed by independent t-test. Main effect of maternal diet ****p < 0.0001; ***p < 0.001; *p < 0.05

Weanlings: Body weight and Retroperitoneal fat (RP) were analysed by two-way ANOVA. *p < 0.01 main effect of maternal diet; ^#^*p* < 0.01 main sex effect

7 and 14 weeks: Body weight and RP fat (14 weeks only) were analysed by three-way ANOVA. *p < 0.05 main effect of maternal diet;^##^*p* < 0.01 main effect of sex; ^ϮϮ^*p* < 0.01 main effect of postnatal diet

Postweaning group names denote maternal and postweaning offspring diets, respectively; for example, ‘ChowCaf’ rats were from chow dams and switched to Caf postweaning

Mothers body weight from diet day 30 to the end of lactation was analysed by two-way repeated measures ANOVA. Caf diet consumption significantly increased dams body weight across time (main effect of maternal diet, *F* = 19.87, *p* < 0.001); and main effect of time (*F* = 283.5, *p* < 0.0001) with significant interaction between maternal diet and time (*F* = 38.46, *p* < 0.0001) (Table 1). Retroperitoneal (RP) fat in Caf mothers was significantly heavier than Chow mothers (t = 2.20, *p* < 0.05). For weanlings, maternal diet and sex did not affect body weight. Interestingly, RP fat mass differed according to maternal diet (main effect of maternal diet, *F* = 21.8, *p* < 0.01) and sex (main effect of sex, *F* = 11.4, *p* < 0.01), however there was no significant maternal diet × sex interaction. At 7 weeks, body weight differed according to postnatal diet (main effect of postnatal diet, *F* = 70.0, *p* < 0.01) and sex (main effect of sex, *F* = 589.6, *p* < 0.01). Maternal diet did not affect body weight nor was there any maternal diet, postnatal diet and sex interactions. At 14 weeks, significant sex × maternal diet interaction on RP fat (*F* = 6.17, *p* < 0.05) and body weight (*F* = 4.3, *p* < 0.05) indicated that maternal diet differentially affected males and females. Also, there was a significant sex × postnatal diet interaction (*F* = 31.66, *p* < 0.01) for RP fat mass. However, there was no significant three-way (maternal diet × postnatal diet × sex) or two-way (maternal diet × postnatal diet) interaction on body weight and RP fat.

For additional phenotypic data of mothers and offspring, overall macronutrient intake and 24 h food consumption in the dams, see the related study [17] and our previous work [28]. Gut microbial diversity of each group was assessed by 16S rRNA genes from fecal samples collected at times shown in Fig. 1.

### Lasting effects of maternal obesogenic diet consumption on gut microbiota α-diversity in mothers and offspring

**Fig. 2:**
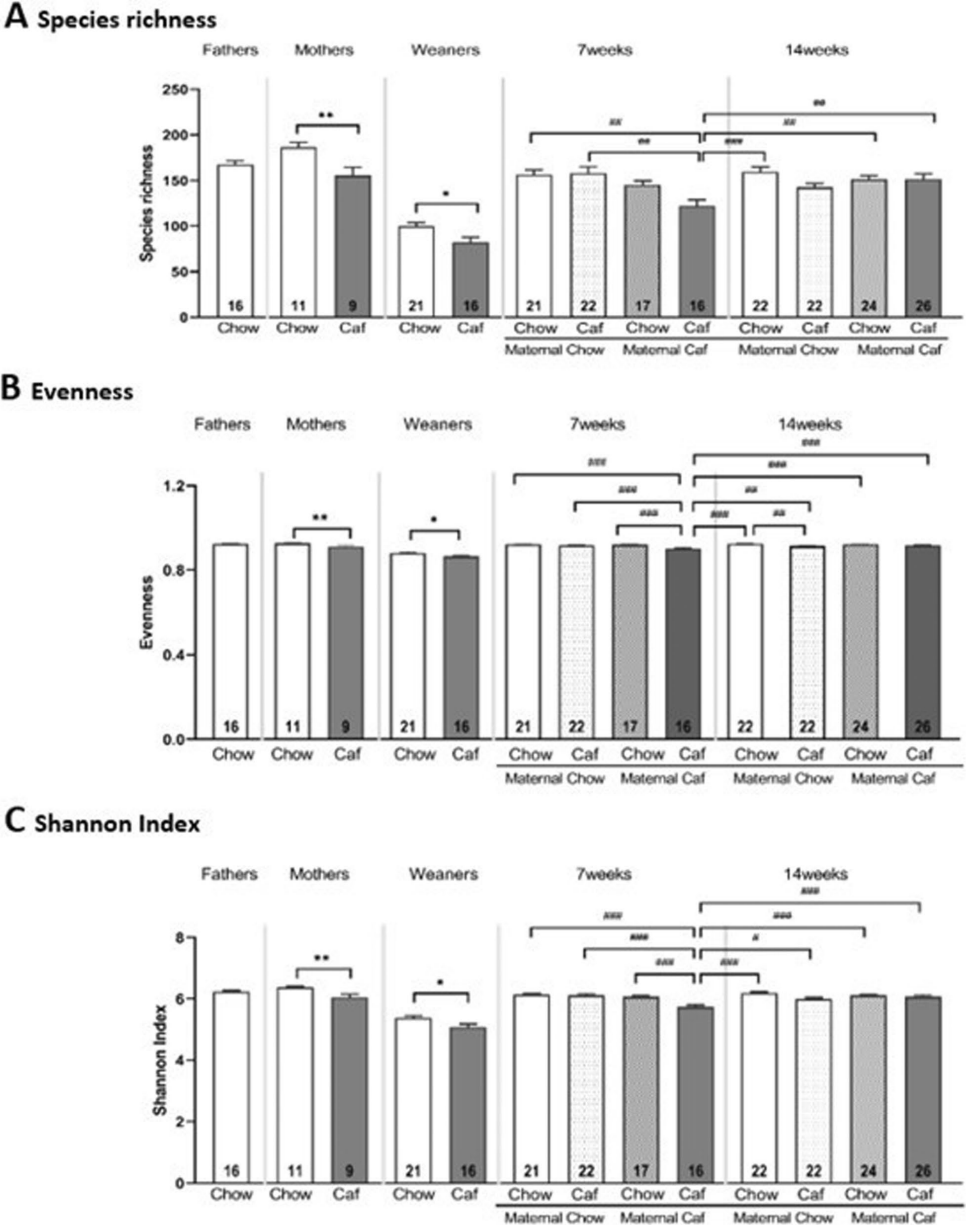
α-diversity across groups and time. **A** Species Richness; **B** Evenness; and **C** Shannon Index. **p < 0.01 independent t test; *p < 0.05 main effect of maternal diet. ^#^*p* < 0.05; ^##^*p* < 0.01; ^###^*p* < 0.001 by Post hoc comparisons. Data are displayed as mean ± SEM. Chow: chow diet; Caf: Cafeteria diet; ChowChow: maternal chow diet and postnatal chow diet; ChowCaf: maternal chow diet and postnatal Caf diet; CafChow: maternal Caf diet and postnatal chow diet; CafCaf: maternal Caf diet and postnatal Caf diet

We first examined α-diversity measures across groups and time. Caf diet consumption significantly depleted species richness, evenness and the Shannon diversity index in Caf mothers (t = 3.05, *p* < 0.01, Fig. 2A; t = 3.84, *p* < 0.01, Fig. 2B; and t = 3.42, *p* < 0.01, Fig. 2C respectively).

At weaning, two-way ANOVA (maternal diet × sex) indicated that maternal Caf diet consumption significantly reduced species richness (*F*_(1,32)_ = 7.475, *p* = 0.01), evenness (*F*_(1,32)_ = 7.347, *p* = 0.011) and the Shannon diversity index (*F*_(1,32)_ = 8.290, *p* = 0.007) regardless of offspring sex, with no significant interactions between maternal diet and sex (Fig. 2A–C). There was no significant cage effect on weaner’s species richness, evenness and Shannon index.

Adult offspring microbiota composition at 7- and 14-week timepoints was analyzed by 2 (maternal diet) × 2 (postnatal diet) × 2 (time) × 2 (sex) factorial ANOVA. A significant three-way interaction between maternal diet, postnatal diet and time were found for species richness (*F*_(1,161)_ = 7.550, *p* = 0.007), evenness (*F*_(1,161)_ = 14.217, *p* < 0.001) and Shannon index (*F*_(1,161)_ = 12.547, *p* = 0.001), while offspring sex did not interact with these variables (Additional file 1: Fig. S1).

To clarify the source of these interactions, we compared the effects of Caf diet on offspring α-diversity measures over time using post hoc comparisons applying the Bonferroni correction. Species richness was significantly reduced in the CafCaf group at 7 weeks compared with ChowChow group at 7 and 14 weeks (*p* < 0.01 and *p* < 0.001 respectively) and CafCaf group at 14 weeks (*p* < 0.01). By 14 weeks, intriguingly, species richness in CafCaf group was no longer different from other groups (Fig. 2A). Similar effects were seen for evenness and Shannon index measures, which were both significantly reduced in the CafCaf group at 7 weeks compared to ChowChow group at 7 and 14 weeks and CafCaf group at 14 weeks. At 14 weeks, evenness and Shannon diversity did not differ between groups (Fig. 2B–C). Finally, we entered cage as a covariate in the three-way analyses. For 7 and 14 week measures, there was some evidence that species richness differed between cages (*p* = 0.03) but critically, this factor did not interact with maternal and offspring diet factors (all *ps* > 0.05), and thus did not appear to determine the results of interest.

Next, we examined the effect of diet switch (maternal diet ≠ postnatal diet) on offspring α diversity measures over time. Offspring from Caf dams switched to chow at weaning (CafChow group) did not differ in α diversity measures (species richness, evenness and Shannon index) relative to the ChowChow group at 7 and 14 weeks (Fig. 2A–C). Likewise, offspring from chow dams switched to Caf (ChowCaf group) did not differ in α-diversity measures from ChowChow group at 7 and 14 weeks. Evenness and Shannon index in the ChowCaf group at 14 weeks was significantly increased compared with the CafCaf group at 7 weeks (Fig. 2B–C).

### β-diversity of gut microbiota shifted over time in a host age-dependent manner

**Fig. 3:**
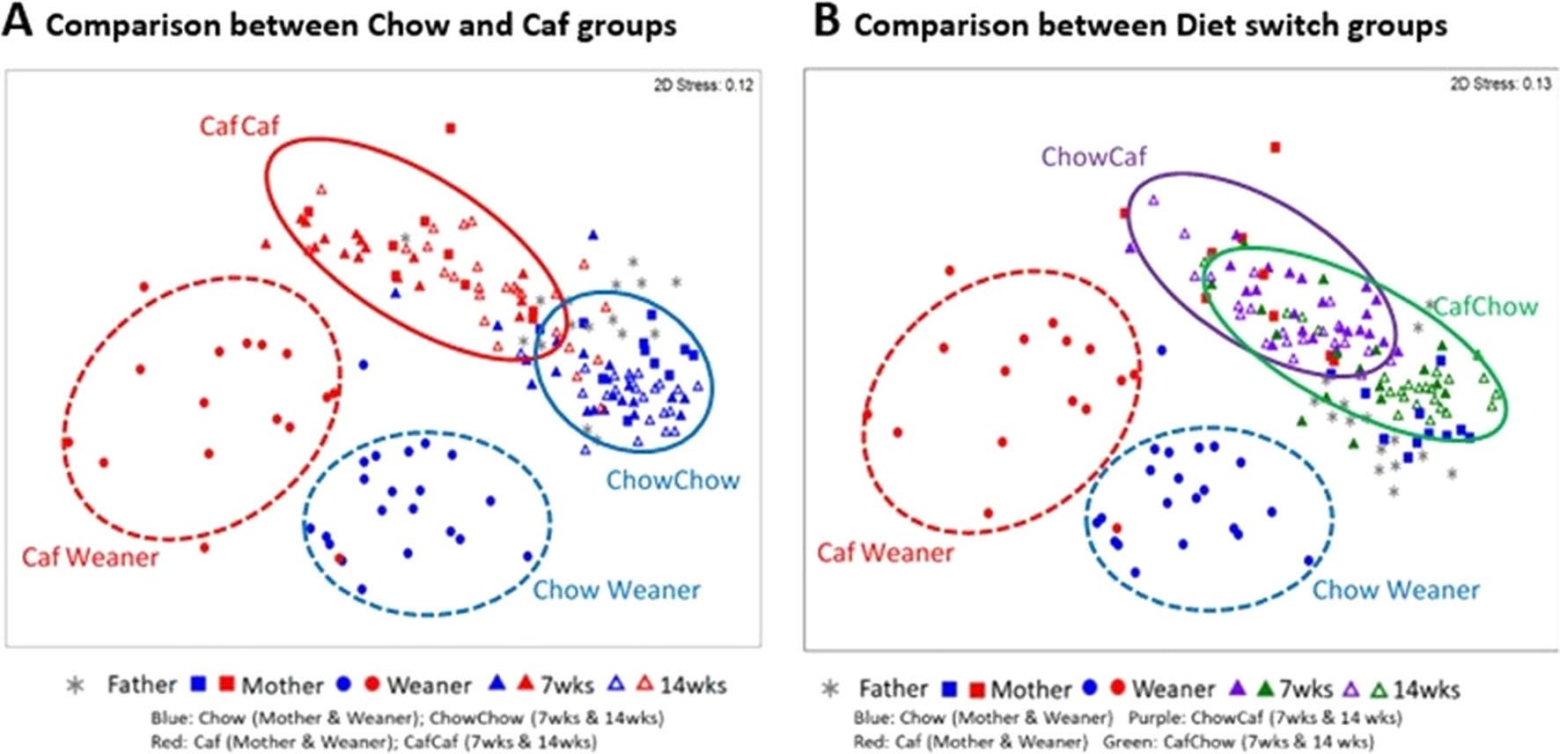
β-diversity. Non-metric multidimensional scaling (NMDS) plots following square root transformation and Bray–Curtis resemblance of relative abundance data at the OTU level. **A** NMDS for Weaner (Chow or Caf), 7wks (ChowChow or CafCaf) and 14wks (ChowChow or CafCaf); Father (Chow only), Mother (Chow or Caf) are also shown. **B** NMDS for Weaner (Chow or Caf), 7wks (ChowCaf or CafChow) and 14 wks (ChowCaf or CafChow); Father (Chow only), Mother (Chow or Caf) are also shown. Chow: chow diet; Caf: Cafeteria diet; ChowChow: maternal chow diet and postnatal chow diet; ChowCaf: maternal chow diet and postnatal Caf diet; CafChow: maternal Caf diet and postnatal chow diet; CafCaf: maternal Caf diet and postnatal Caf diet

We first examined the impact of chow and Caf diet on gut microbial communities of mothers, and offspring at weaning, 7 and 14 weeks; all groups clustered differently regardless of diet type at the OTU level, as indicated by non-metric multidimensional scaling (NMDS) plots (Fig. 3A and B; and Additional file 3: Fig. S3A–D) and PCO (Additional file 2: Fig. S2). PERMANOVA analyses (999 permutations) confirmed significant differences in β-diversity between Chow and Caf mother (*F*_(1,18)_ = 6.7575, *p* = 0.001) and between Chow weaner and Caf weaner (F_(1,33)_ = 6.4525, *p* = 0.001) (Table 2). There was no significant interaction between maternal diet and sex on β-diversity of weanlings.

**Table 2:**
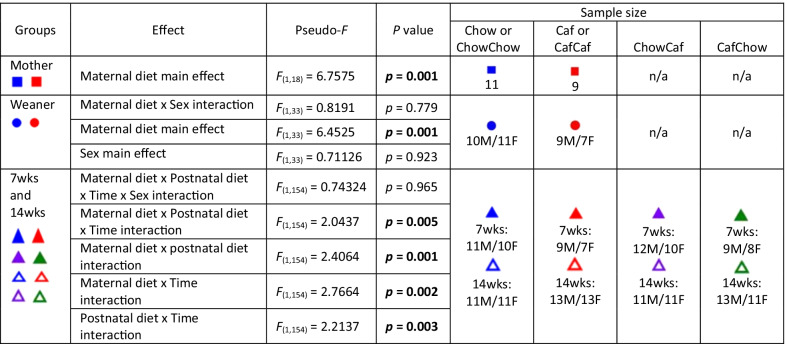
Summary of PERMANOVA analyses

Symbol: blue square (chow mother); red square (caf mother); blue circle (chow weaner); red circle (chow weaner); blue triangle (chowchow at 7wks); red triangle (cafcaf at 7wks); purple triangle (chowcaf at 7wks); green triangle (cafchow at 7wks). Note open symbols of the same color indicate 14 wk data

Results from PERMANOVA (999 permutations) analyses are summarised in Table 2. For β-diversity of offspring at 7 and 14 weeks, we performed PERMANOVA using a 2 (maternal diet) × 2 (postnatal diet) × 2 (time) × 2 (sex) design. The analysis indicated a significant 3-way interaction between maternal diet, postnatal diet and time (F_(1,154)_ = 2.0437, *p* = 0.005) (Table 2). PERMDISP (variation of Bray–Curtis similarities) confirmed no systematic differences in sample dispersion (Mother: *F*_(1,18)_ = 0.0267, *p* = 0.889; Weaner: *F*_(1,35)_ = 2.213, *p* = 0.169; 7 and 14 weeks: *F*_(7, 162)_ = 1.444, *p* = 0.262). Pairwise comparisons between offspring groups at 7 and 14 weeks confirmed that microbiota composition significantly differed between all groups (largest *p* < 0.033).

### Continuous Caf diet consumption altered abundance of OTUs

**Fig. 4:**
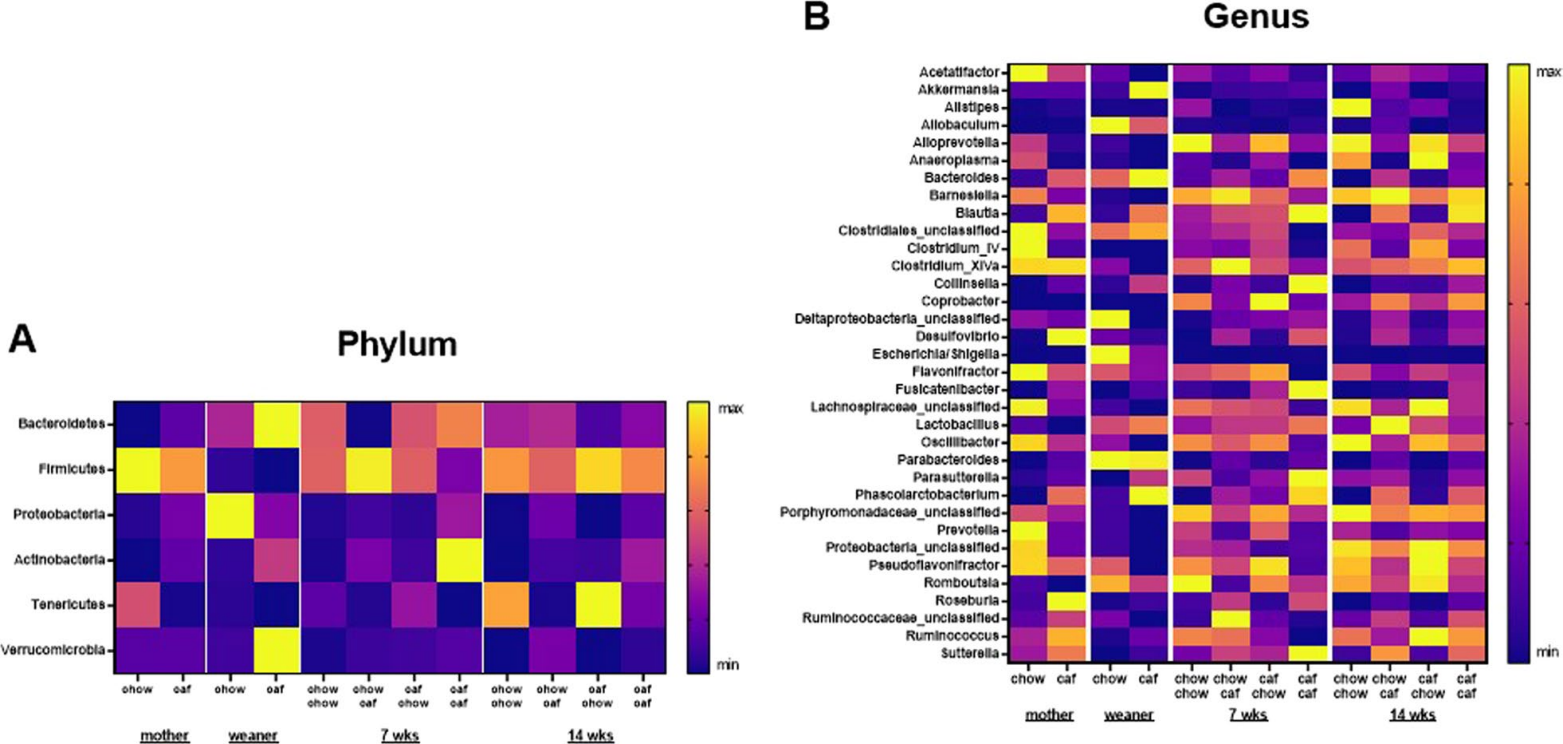
Representative taxa on the phylum and genus levels in each group. Microbial taxa top 100 OTUs were selected to generate heat maps on phylum level **(A)** and genus level **(B)**. The relative abundance of phylum and genus levels were normalised by row

Figure 4 shows representative taxa on phylum (A) and genus (B) levels in each group. The top 100 OTUs were selected to generate heat maps on phylum (A) and genus level (B), and the heatmaps were normalised by row. At phylum level, *Firmicutes* was more abundant in both chow and Caf mothers compared with *Bacteroidetes;* on the other hand, *Bacteroides* was more abundant in both chow and Caf weanlings compared with *Firmicutes*. *Proteobacteria* and *Verrucomicrobia* were highly abundant in chow and Caf weanlings respectively.

**Fig. 5:**
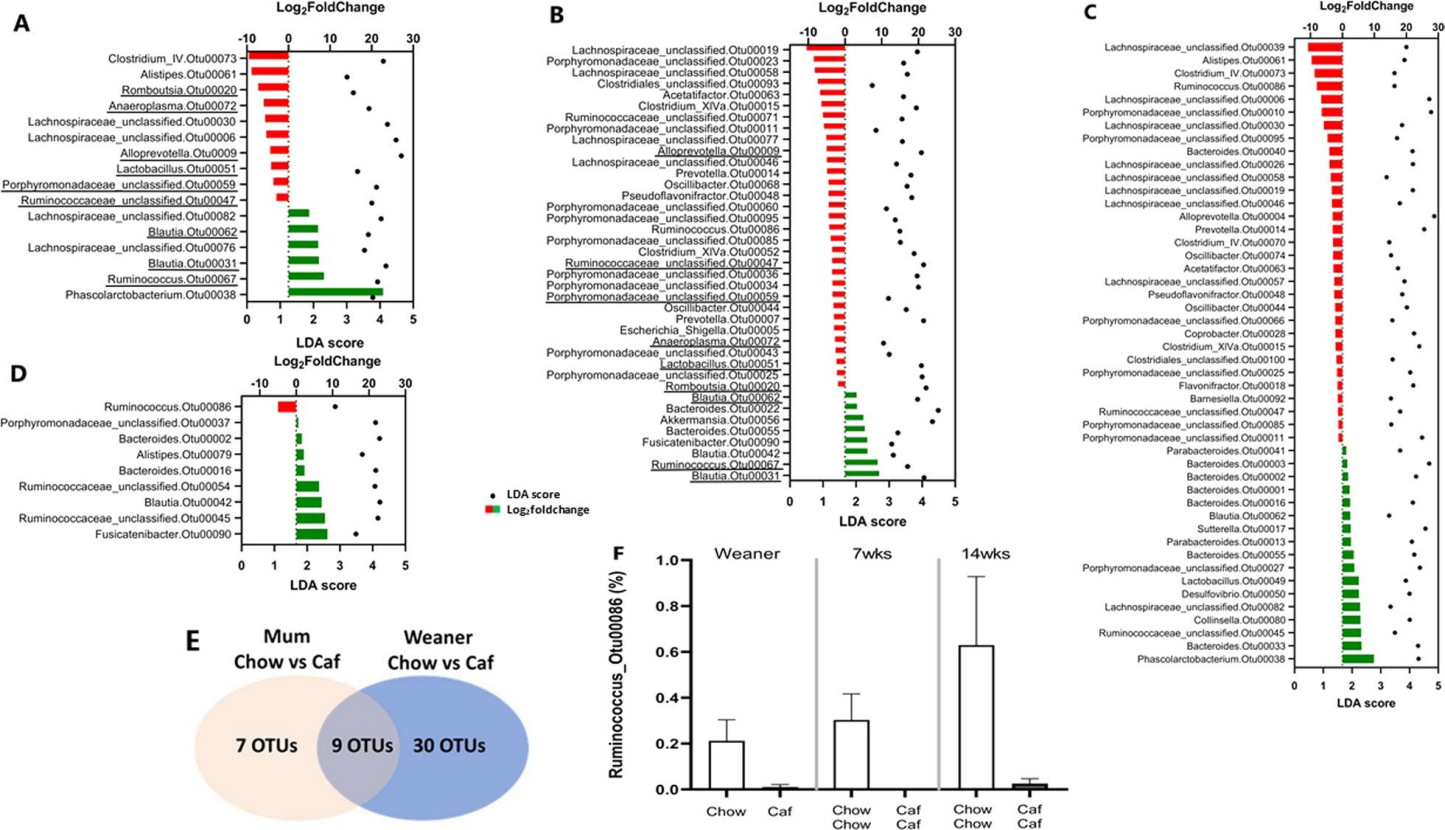
OTUs significantly altered between groups. Microbial taxa among the top 100 OTUs identified to significantly differ in abundance between **A** mothers (Chow vs. Caf); **B** weanlings (Chow vs Caf), underlined OTUs indicate 9 common taxa between mothers and weanlings; **C** offspring at 7 weeks (ChowChow vs CafCaf); and **D** offspring at 14 weeks (ChowChow vs. CafCaf) by DESeq2 (*padj* < 0.05) and LEfSe (LDA Score > 2.0, *p* < 0.05). In DESeq2, negative (red) Log2foldchange value denotes decreased abundance and positive (green) Log_2_foldchange value denotes increased abundance in **A** Caf mother; **B** Caf weaner; **C** 7 week offspring in CafCaf group; and **D** 14 week offspring in CafCaf group. **E** Differential OTUs 7 OTUs and 30 OTUs were uniquely altered in Mothers and Weanlings by maternal Caf diet, with 9 common OTUs in Caf Mothers and Caf Weanlings. **F** Relative abundance of *Ruminococcus*_Otu00086 in 3-, 7- and 14-week offspring fed Caf diet compared with offspring fed chow diet. Note differences were significant using both LEfSe and DESeq2

Next, DESeq2 and LEfSe analyses were used to identify OTUs that were differentially enriched or depleted by Caf diet exposure between groups and across time. To assess the effects of diet exposure, we selected OTUs only if the adjusted *p* value was < 0.05 from both DESeq2 and LEfSe analyses. Enriched/depleted OTUs meeting these criteria were then assessed and the top 100 OTUs were selected. In mothers, a total of 16 OTUs (10 depleted, 6 enriched) were significantly affected by Caf diet (Fig. 5A). Maternal Caf diet was associated with depletion of several OTUs in *Lactobacillus* and *Alloprevotella* genera, while several OTUs in the *Blautia* and *Ruminococcus* genera were enriched. At weaning, 8 OTUs were enriched while 31 were depleted in the offspring of Caf dams relative to those from chow dams (Fig. 5B). There were 9 OTUs commonly affected by maternal Caf diet in both mothers and weanlings (see underlined taxa in Fig. 5A and B; E). At 7 weeks, 48 OTUs were significantly affected in CafCaf compared to ChowChow offspring, while at 14 weeks, 9 OTUs differed significantly in abundance in CafCaf compared to ChowChow offspring (Fig. 5C and D). The relative abundance of *Ruminococcus*_*Otu00086* was consistently and significantly decreased in CafCaf offspring at 3, 7 and 14 weeks (Fig. 5F).

We further explored OTUs affected by postnatal diet switch by comparing ChowChow and ChowCaf groups. There were 22 OTUs at 7 weeks and 45 OTUs at 14 weeks that significantly differed in abundance between ChowCaf and ChowChow groups by Log_2_foldchange and LDA score. Fifteen OTUs were commonly affected at both 7 and 14 weeks, characterised by significantly depleted abundance of *Alistipes*, *Alloprevotella* and *Prevotella* genera and increased abundance of *Phascolarctobacterium* and *Ruminococcaceae* genera (Table 3). On the other hand, 32 OTUs at 7 weeks and 33 OTUs at 14 weeks significantly differed in abundance between CafChow and CafCaf groups; 14 OTUs were altered at both timepoints, with decreased abundance of *Ruminococcus* and *Lachnospiraceae* genera and increased abundance of *Lactobacillus* and *Collinsella* genera in the CafCaf group (Table 4). For the comparison between ChowChow and CafChow groups, no OTUs survived FDR correction.

**Table 3:** Common OTUs altered significantly in ChowCaf compared with ChowChow offspring at both 7 and 14 weeks

	7 weeks		14 weeks	
	Log_2_fold change	LDA score	Log_2_fold change	LDA score
Alloprevotella_Otu00009	− 2.3083	4.3576	− 1.8990	4.3127
Parabacteroides_Otu00013	1.5708	3.9121	2.2776	3.8774
Prevotella_Otu00014	− 2.3233	4.5135	− 1.2585	4.3395
Bacteroides_Otu00016	1.1072	3.8747	2.6325	4.0991
Porphyromonadaceae_unclassified_Otu00025	− 1.5090	4.0279	− 2.0970	4.2307
Porphyromonadaceae_unclassified_Otu00027	2.3246	4.0002	2.5798	3.8652
Phascolarctobacterium_Otu00038	8.0093	3.8860	11.0405	4.1459
Parabacteroides_Otu00041	1.5970	3.8413	2.3272	3.8727
Ruminococcaceae_unclassified_Otu00045	8.7606	4.3946	8.3633	4.1660
Lactobacillus_Otu00049	4.2348	3.6530	3.3537	3.7545
Desulfovibrio_Otu00050	4.2064	3.8123	3.5553	3.8471
Ruminococcaceae_unclassified_Otu00054	6.9617	3.7397	4.3344	3.4625
Alistipes_Otu00061	− 5.0765	3.8286	− 6.2610	4.2964
Blautia_Otu00062	3.2248	3.5987	5.3293	4.0366
Lachnospiraceae_unclassified_Otu00082	5.6708	3.6282	6.0649	3.7067

**Table 4:** Common OTUs altered significantly in CafCaf compared with CafChow offspring at both 7 and 14 weeks

	7 weeks			14 weeks	
	Log_2_fold change	LDA score		Log_2_fold change	LDA score
Bacteroides_Otu00001	2.0001	5.3396		1.1861	4.9270
Bacteroides_Otu00003	2.3147	4.6861		1.7198	4.5564
Lachnospiraceae_unclassified_Otu00006	− 6.0196	4.4853		− 2.6915	4.8489
Parabacteroides_Otu00013	2.3522	4.0860		2.6392	3.8844
Bacteroides_Otu00016	2.2236	4.1197		1.9798	3.8972
Sutterella_Otu00017	2.2638	4.5561		1.7500	4.3274
Porphyromonadaceae_unclassified_Otu00027	1.7907	4.3650		3.5524	4.2215
Lachnospiraceae_unclassified_Otu00030	− 5.8341	3.6741		− 2.8812	4.3733
Lachnospiraceae_unclassified_Otu00046	− 4.0217	3.9064		− 2.0278	4.1302
Pseudoflavonifractor_Otu00048	− 2.8019	3.8461		− 1.1043	3.8574
Lactobacillus_Otu00049	6.6289	3.8744		1.7349	3.8290
Oscillibacter_Otu00044	− 2.2025		3.9289	− 2.0080	3.5923
Collinsella_Otu00080	4.2399		4.0121	8.2465	3.5511
Ruminococcus_Otu00086	− 7.7353		3.4690	− 3.5652	3.4270

### Contribution of maternal and paternal gut microbial community to composition of offspring gut microbiota

**Fig. 6:**
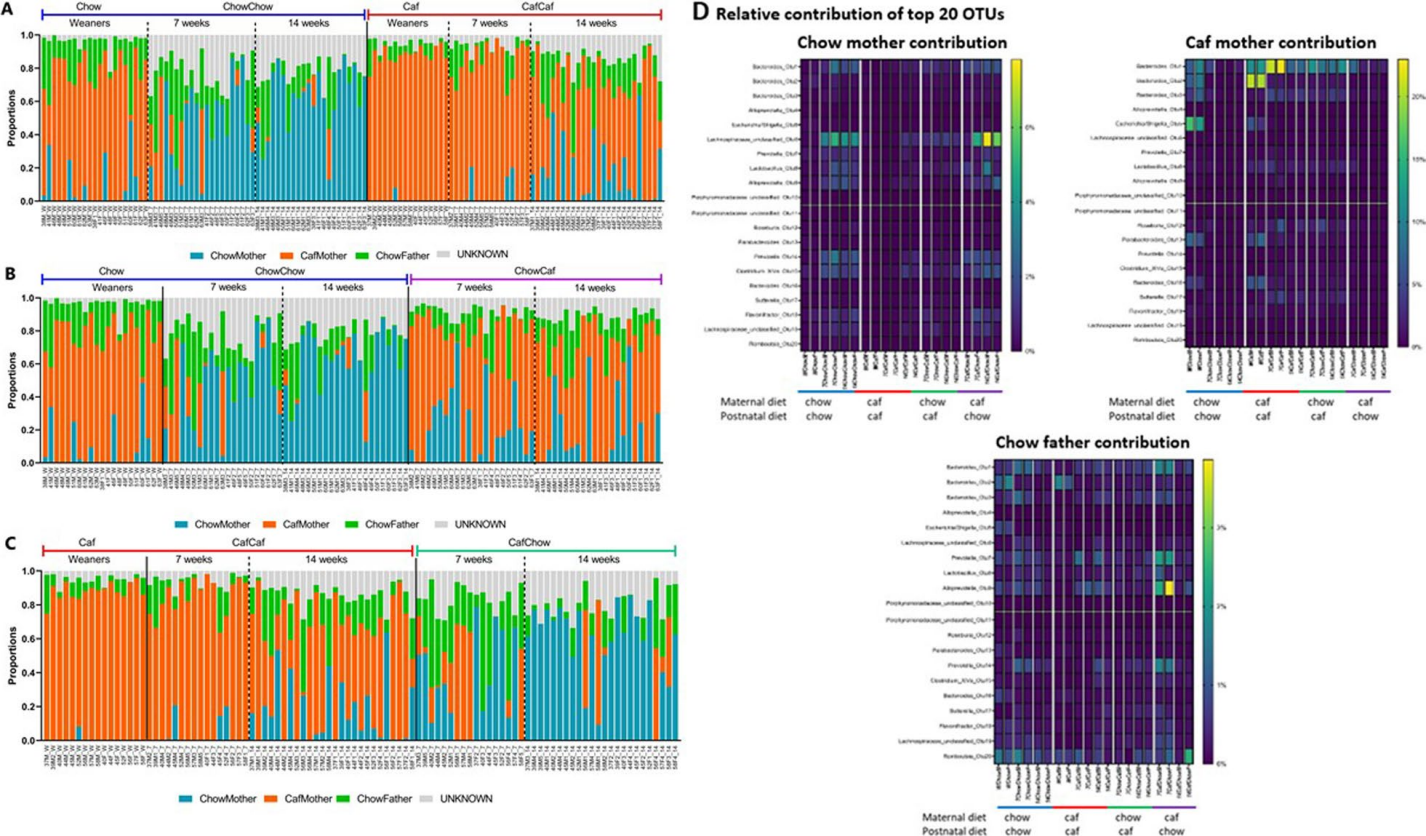
SourceTracker analyses. Chow mothers, Caf mothers and Chow fathers were pooled separately and computed as sources that can contribute to offspring gut microbial communities. **A** shows offspring consuming same diet as mother; Chow and Caf weanlings, ChowChow and CafCaf offspring at 7 and 14 weeks. **B** compares offspring of Chow mothers; Chow weanlings, ChowChow and ChowCaf offspring at 7 and 14 weeks. **C** compares offspring of Caf mothers; Caf weanlings, CafChow and CafCaf offspring at 7 and 14 weeks. Individual rats are indicated on X axis (number indicates mother id, M or F indicates male or female). **D** Relative contribution of top 20 OTUs of Chow mother, Caf mother and Chow father gut microbiota to offspring gut microbiota

We used SourceTracker [26] to assess any contributions of maternal and paternal gut microbial community to offspring. This revealed differential contributions of species associated with the gut microbiota from Chow mothers, Caf mothers, and fathers, to offspring gut microbiota. Pooled Chow mothers, Caf mothers and fathers were treated as separate sources contributing organisms to offspring gut microbial communities at weaning, 7 and 14 weeks of age (Fig. 6A–C). SourceTracker suggested that the microbiota composition of Caf mothers made a larger contribution to both Chow and Caf weanlings’ gut microbiota than did that of Chow mothers (Additional file 4: Fig. S4). Chow mothers’ contribution to offspring microbiota increased upon switching to postnatal chow diet over time, with offspring showing an increase in chow diet associated species. To a lesser extent, a similar trend was observed for offspring in the CafCaf group. On the other hand, Caf mothers’ contribution to offspring decreased upon switching to postnatal chow diet over time, and this was also observed in the CafCaf group. Paternal contribution to offspring gut microbiota was greatest in weanlings and decreased over time across groups (Additional file 4: Fig. S4).

Figure 6D shows the contributions of top 20 OTUs from Chow and Caf mothers and fathers to offspring gut microbiota. SourceTracker indicated differential transmission according to maternal diet; for Caf mother microbiota, this tended towards *Bacteroides*_Otu00001 and *Bacteroides*_Otu00002, while the contribution of Chow mother microbiota tended towards *Lachnospiraceae_unclassified*_Otu00006, *Prevotella*_Otu00007, *Lactobacillus*_Otu00008 and *Alloprevotella*_Otu00009 (Fig. 5D). There was a greater influence of Caf mother (up to 22%) compared with Chow mother (up to 7%) and father (up to 4%) contribution to offspring (Fig. 6D). FASTA sequences of these OTUs were blasted against whole genome shotgun contigs (wgs) to identify bacterial species in the Basic Local Alignment Search Tool (BLAST) (https://blast.ncbi.nlm.nih.gov/Blast.cgi). The BLAST search showed that *Bacteroides*_Otu00001 had similarity to *Bacteroides vulgatus* (*Phocaeicola vulgatus*) (98.8%), *Bacteroides*_Otu00002 had similarity to *Bacteroides acidifaciens* (98.8%), *Lactobacillus*_Otu00008 had similarity to *Lactobacillus murinus (Ligilactobacillus murinus)* (98.4%) and *Alloprevotella*_Otu00009 had similarity to *Alloprevotella rava* (88.4%), whereas BLAST search for *Lachnospraceae*_*unclassified*_Otu00006 and *Prevotella*_Otu00007 did not result in identifiable species.

## Discussion

Our study showed that the development of gut microbiota from weaning to adulthood in rats was characterized by increased α-diversity and reduced dissimilarity in β-diversity; changes that rapidly occurred after weaning onto solid food. These results are consistent with studies in people showing that the cessation of breast milk triggered infant gut microbiota development [29, 30], indicating a similar shift in the development of infant gut microbiota to adult-like gut microbiota across different species. Furthermore, we showed that maternal and postnatal consumption of a Caf diet comprised of palatable, processed foods eaten by people, reduced α diversity and altered β-diversity compared with maternal consumption of chow. On the other hand, a maternal Caf diet did not appear to prevent the age-associated increase in α diversity and reduction in β-diversity dissimilarity associated with the shift of offspring gut microbiota towards an adult-like profile.

At weaning, the gut microbial communities of offspring of Chow and Caf dams showed dramatically reduced α-diversity and altered β-diversity relative to their mothers and older offspring (7 and 14 weeks). At 7 weeks of age, α-diversity was suppressed in the CafCaf group compared with the ChowChow, ChowCaf and CafChow groups, suggesting an additive effect of maternal and postweaning exposure to Caf diet. The overall gut microbial composition (β-diversity) of 7 week-old ChowChow and CafCaf offspring clustered close to, but were nonetheless significantly different from, their respective mothers. In contrast, by 14 weeks of age, there was no difference in species richness, evenness and Shannon’s index across the four offspring groups. However, it is possible that the difference in gut microbial composition at 7 weeks of age might modulate the subsequent programming of offspring health in later life. This may suggest adaptation of offspring gut microbiota to different types of diet, however, there are complex interactions between maternal diet and postnatal diet over time. It is also possible that development of the gastrointestinal tract in offspring [31] might have contributed to the absence of group differences in later life, hence further investigation is warranted.

β-diversity of offspring gut microbiota exhibited greater similarity to the adult-like gut microbiota over time, both for groups whose postweaning diet was consistent with that of the mother (ChowChow and CafCaf) and for those whose postweaning diet differed from their mother (ChowCaf and CafChow groups). The trend of offspring gut microbiota maturation in this study, an increase in α-diversity and a decrease in dissimilarity of β-diversity is consistent with work by Bäckhed et al. (2015) examining the human gut microbiota in mother and infant dyads [29]. In addition, we show for first time that the developmental trajectory of infant gut microbiota was not affected by the type of maternal and postnatal diets. Maternal factors including the use of antibiotics and other medications, birth mode (vaginal vs C-section), diet and stress have been reported to influence the offspring gut microbiota [1]. However, there is still sparse longitudinal evidence of the impact of these factors on development of the gut microbiota.

Nonetheless, DESeq2 and LEfSe analyses identified multiple OTUs that were reliably altered in the CafCaf relative to the ChowChow group across development. At 7 weeks, the largest number of OTUs were altered in CafCaf group; in total 48; 31 OTUs were depleted and 17 OTUs were enriched. By 14 weeks, the number of altered OTUs in CafCaf group compared with ChowChow group was 9 OTUs in total, of which 1 taxon was depleted and 8 taxa were enriched. Thus fewer OTUs differed between groups over time, underlining an increasing similarity of α-diversity and β-diversity of offspring gut microbiota. On the other hand, the relative abundance of *Ruminococcus*_Otu00086 was consistently depleted in Caf weanlings and in group CafCaf at 7 and 14 weeks, compared with Chow groups. BLAST search with FASTA sequence of Otu_00086 did not identify any similarity to *Ruminococcus* species. Depletion of Otu_00086 was also observed in Caf dams (relative abundance 6.3%) compared with chow dams (42.2%). Together, this may indicate *Ruminococcus* is a potential biomarker for unhealthy dietary intake. Here offspring of Caf dams mirrored the effect of Caf diet on suppressing *Ruminococcus,* the effect that was consistent over time, since the depletion of the genus was observed in offspring gut microbiota from weaning to 14 weeks. In support, the relative abundance of *Ruminococcus*_Otu86 recovered after switching to chow diet. *Ruminococcus* are commensal bacteria and play key roles in plant fiber degradation (resistant starch) and butyrate production [32, 33]. Continuous Caf diet consumption suppressed the abundance of this beneficial bacteria, which has been shown previously to be depleted [34] in Caf fed rats compared with purified high fat diet and ‘western style’ diet fed animals. Our finding is consistent with findings of Sonnenberg et al. [35] describing extinction of taxa in mice consuming a western style diet lacking dietary fibre over multiple generations [35]. Hence, this may suggest that the *Ruminococcus* genera might be affected by highly processed foods containing food additives and insufficient dietary fibre, rather than an effect of the energy density of the food per se [36, 37].

SourceTracker analysis indicated a greater overall contribution of Caf mothers’ microbial community (up to 20%) to that of their offspring than the contribution of Chow mothers’ (up to 8%) of the offspring community. The greater contribution of Caf mothers may be due to higher energy efficiency of representative taxa, however, this needs to be interpreted with caution. Another intriguing finding was the greater contribution of Caf mothers’ microbial community to both Caf and chow weanlings gut microbial community at PND19. Caf mothers’ contribution was characterized by higher relative abundance of *Bacteroides*_Otu00001 (98% similarity to *Bacteoides vulgatus*) and *Bacteroides*_Otu00002 (98% similarity to *Bacteroides acidifaciens*). On the other hand, Chow mothers’ contribution was characterized by higher relative abundance of *Lachnospiraceae_unclassified*_Otu00006, *Prevotella*_Otu00007, *Lactobacillus*_Otu00008 (98% similarity to *Lactobacillus murinus*) and *Alloprevotella*_Otu00009 (88% similarity to *Alloprevotella rava*). Increased relative abundance of *Bacteroides vulgatus* was in line with a higher abundance of this species in high fat diet fed rats in our previous study [38].

Finally, we did not find any evidence that sex interacted with α-diversity and β-diversity measures on the development of offspring gut microbiota in this cohort.

The study has some limitations. As the Caf style diet used mirrors the western diet eaten by people [16], by design, it includes foods containing various additives, preservatives, emulsifiers and colorants which can affect gut microbial diversity, in addition to being low in fibre. Future studies using purified diets could delineate the differential impacts of individual diet components on development of gut microbiota. As offspring gut microbial diversity rapidly changes on introduction of solid foods at weaning, it would be interesting to sample soon after the introduction of solid foods to provide additional insight into microbiota development in early life. This study assessed maternal microbial community based on feces collected from the mother at weaning. Recent evidence indicates that maternal microbial communities fluctuate during gestation [39]. Hence, maternal gut microbiota at weaning might differ from that found pre-mating, during gestation and lactation. Lastly, the paternal microbiota was assessed by sampling feces from fathers at weaning, which may not accurately reflect the paternal microbiota at mating.

## Conclusion

This study using rats showed that cessation of lactation triggered offspring gut microbiota maturation, in line with past human studies. The introduction to solid food drives early life gut microbiota maturation towards adult-like gut microbiota over time by an increase in α-diversity and reduction in dissimilarity of β-diversity. Unhealthy maternal diets can shift β-diversity of the growth trajectory of offspring gut microbiota. Intriguingly, despite identifying several enriched and depleted bacteria in rats exposed to postweaning Caf diet, the overall maturation of gut microbiota in offspring was not affected by diet type. Our results are in line with previous work and underscore the notion that introduction of solid food plays an important role in shaping early life gut microbiota composition.

## Supplementary Information

**Additional file 1: Figure S1**. α-diversity three-way interaction plots. Three-way interaction between maternal diets, postnatal diets and time at 7 and 14 weeks shown in A) Species Richness; B) Evenness; and C) Shannon Index.

**Additional file 2: Figure S2**. β-diversity (PCO).

**Additional file 3: Figure 3**. β-diversity by group. β-diversity by groups were shown, A) Mother; B) Weaner; C) 7weeks; and D) 14weeks.

**Additional file 4: Figure 4**. SourceTracker analysis. Relative contributions to offspring by Chow mothers, Caf mothers and Chow fathers.

**Additional file 5: Table S1**. SourceTracker analysis: Proportions of Chow and Caf mother microbial communities’ contribution to offspring gut microbial composition. Chow mothers, Caf mothers and fathers were pooled separately as environmental sources. Data are displayed as mean ± SEM.

## Data Availability

The sequence data for this study have been deposited in the European Nucleotide Archive (ENA) at EMBL-EBI under accession number PRJEB46141 (https://www.ebi.ac.uk/ena/browser/view/PRJEB46141).

## Abbreviations

ANOVA: Analysis of variance
BLAST: Basic Local Alignment Search Tool
Caf: Cafeteria diet
CafCaf: Maternal caf diet and postnatal caf diet
CafChow: Maternal caf diet and postnatal chow diet
Chow: Standard chow diet
ChowCaf: Maternal chow diet and postnatal caf diet
ChowChow: Maternal chow diet and postnatal chow diet
LDA: Linear Discriminant Analysis
LEfSe: Linear Discriminant Analysis Effect Size
NMDS: Non-metric multidimensional scaling
OTU: Operational taxonomic unit
PERMANOVA: Permutational Multivariate Analysis of Variance
PERMDISP: Permutational Analysis of Multivariate Dispersions
