# Supplementary figures and images for: The influence of maternal unhealthy diet on maturation of offspring gut microbiota in rat
Source: Anim Microbiome. 2022 May 12;4:31. doi: 10.1186/s42523-022-00185-w (PMC9102338; doi:10.1186/s42523-022-00185-w)

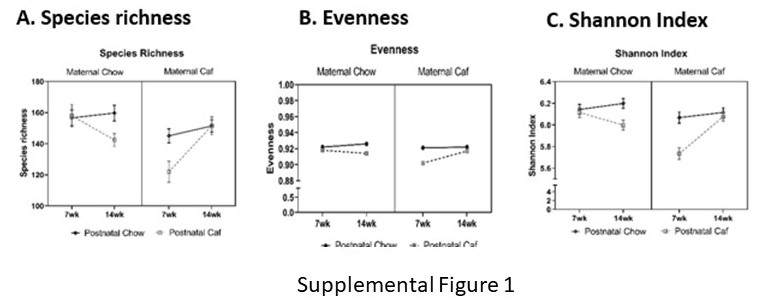

Supplement: Supplementary file 1 — Additional file 1: Figure S1. α-diversity three-way interaction plots. Three-way interaction between maternal diets, postnatal diets and time at 7 and 14 weeks shown in A) Species Richness; B) Evenness; and C) Shannon Index. [file 42523_2022_185_MOESM1_ESM.jpg]

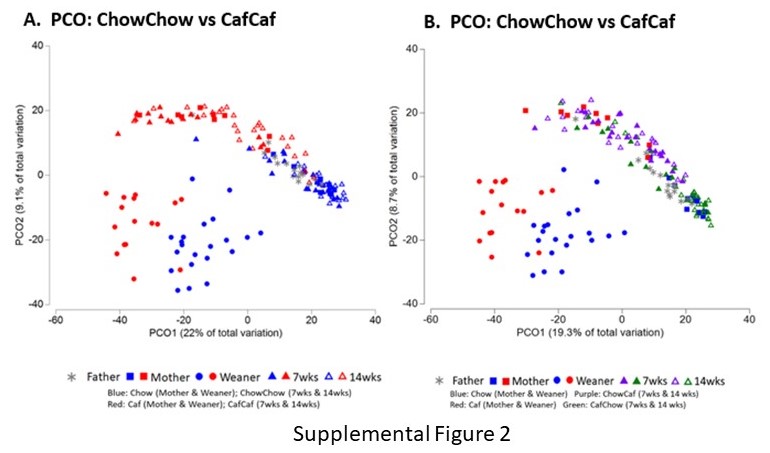

Supplement: Supplementary file 2 — Additional file 2: Figure S2. β-diversity (PCO). [file 42523_2022_185_MOESM2_ESM.jpg]

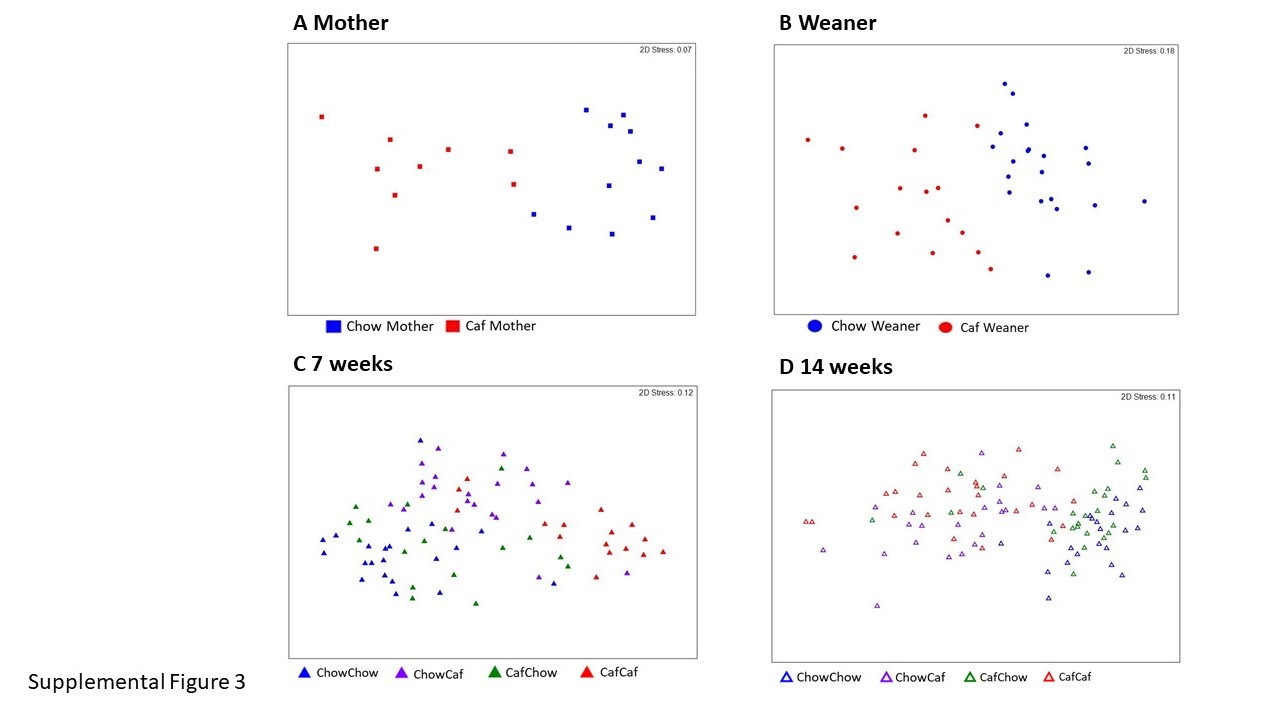

Supplement: Supplementary file 3 — Additional file 3: Figure 3. β-diversity by group. β-diversity by groups were shown, A) Mother; B) Weaner; C) 7weeks; and D) 14weeks. [file 42523_2022_185_MOESM3_ESM.jpg]

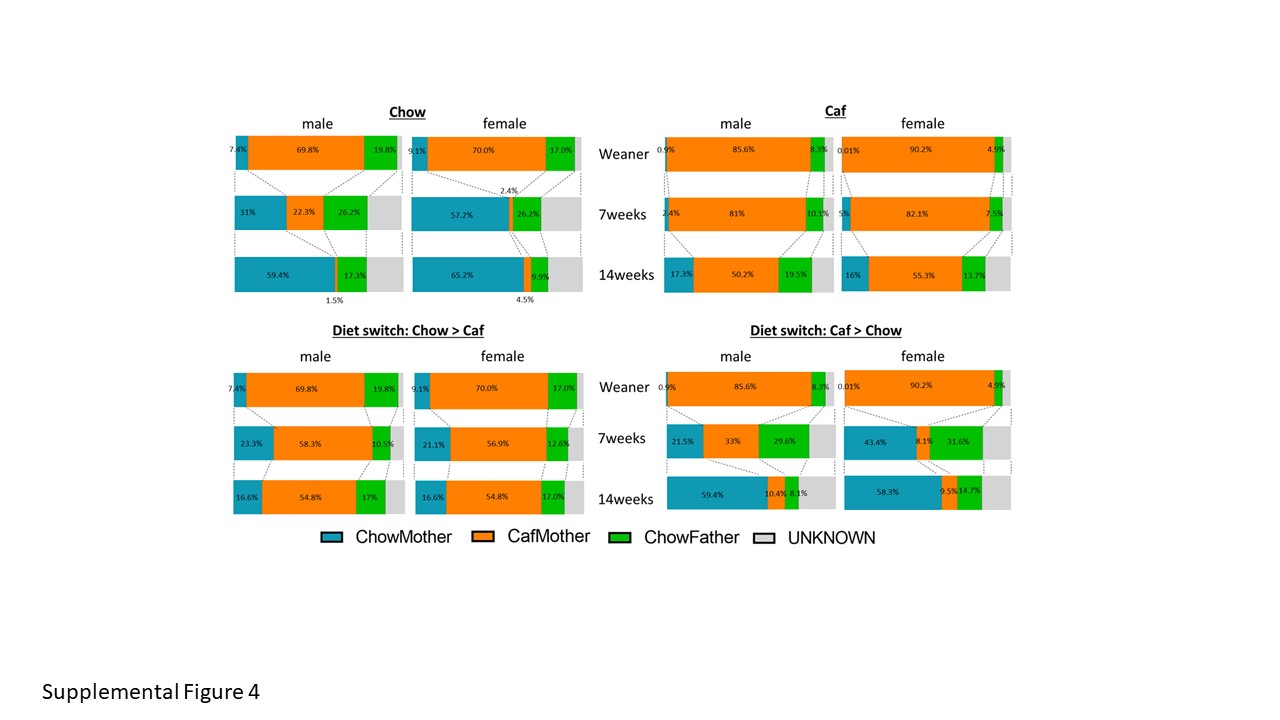

Supplement: Supplementary file 4 — Additional file 4: Figure 4. SourceTracker analysis. Relative contributions to offspring by Chow mothers, Caf mothers and Chow fathers. [file 42523_2022_185_MOESM4_ESM.jpg]
