# Supplementary material for: The influence of maternal unhealthy diet on maturation of offspring gut microbiota in rat
Source: Anim Microbiome. 2022 May 12;4:31. doi: 10.1186/s42523-022-00185-w (PMC9102338; doi:10.1186/s42523-022-00185-w)
Supplement: Supplementary file 5 — Additional file 5: Table S1. SourceTracker analysis: Proportions of Chow and Caf mother microbial communities’ contribution to offspring gut microbial composition. Chow mothers, Caf mothers and fathers were pooled separately as environmental sources. Data are displayed as mean ± SEM. [file 42523_2022_185_MOESM5_ESM.docx]

Additional file 5_Supplemental table 1

| Groups | Diet | Sex (n) | Chow mother  Mean (SEM) | Caf mother  Mean (SEM) | Father  Mean (SEM) | Unknown  Mean (SEM) |
| --- | --- | --- | --- | --- | --- | --- |
| Weaner | Chow | Male (10) | 0.074 (0.038) | 0.698 (0.065) | 0.198 (0.036) | 0.030 (0.007) |
|  | Chow | Female (11) | 0.091 (0.048) | 0.700 (0.071) | 0.170 (0.037) | 0.039 (0.018) |
|  | Caf | Male (9) | 0.009 (0.009) | 0.856 (0.022) | 0.083 (0.021) | 0.052 (0.011) |
|  | Caf | Female (7) | 0.0001 (0.0001) | 0.902 (0.016) | 0.049 (0.016) | 0.049 (0.010) |
| 7 weeks | ChowChow | Male (11) | 0.310 (0.085) | 0.223 (0.076) | 0.262 (0.056) | 0.205 (0.029) |
|  | ChowChow | Female (10) | 0.572 (0.062) | 0.024 (0.016) | 0.164 (0.047) | 0.239 (0.035) |
|  | ChowCaf | Male (12) | 0.233 (0.066) | 0.583 (0.079) | 0.105 (0.017) | 0.079 (0.012) |
|  | ChowCaf | Female (10) | 0.211 (0.061) | 0.569 (0.079) | 0.126 (0.046) | 0.093 (0.016) |
|  | CafChow | Male (9) | 0.215 (0.070) | 0.330 (0.089) | 0.296 (0.051) | 0.159 (0.029) |
|  | CafChow | Female (8) | 0.434 (0110) | 0.081 (0.067) | 0.316 (0.097) | 0.169 (0.024) |
|  | CafCaf | Male (9) | 0.0.024 (0.023) | 0.810 (0.044) | 0.101 (0.032) | 0.064 (0.014) |
|  | CafCaf | Female (7) | 0.050 (0.033) | 0.821 (0.080) | 0.075 (0.037) | 0.054 (0.016) |
| 14 weeks | ChowChow | Male (11) | 0.594 (0.058) | 0.015 (0.008) | 0.173 (0.042) | 0.218 (0.019) |
|  | ChowChow | Female (11) | 0.652 (0.062) | 0.045 (0.031) | 0.099 (0.042) | 0.204 (0.022) |
|  | ChowCaf | Male (11) | 0.166 (0.068) | 0.548 (0.078) | 0.170 (0.045) | 0.114 (0.013) |
|  | ChowCaf | Female (11) | 0.182 (0.072) | 0.565 (0.073) | 0.137 (0.024) | 0.117 (0.013) |
|  | CafChow | Male (13) | 0.594 (0.062) | 0.104 (0.068) | 0.081 (0.020) | 0.221 (0.020) |
|  | CafChow | Female (11) | 0.583 (0.078) | 0.095 (0.058) | 0.147 (0.041) | 0.175 (0.027) |
|  | CafCaf | Male (13) | 0.173 (0.052) | 0.502 (0.092) | 0.195 (0.040) | 0.129 (0.017) |
|  | CafCaf | Female (13) | 0.160 (0.053) | 0.553 (0.078) | 0.137 (0.021) | 0.151 (0.020) |

Chow mothers, Caf mothers and fathers were pooled separately as environmental sources. Data are displayed as mean ± SEM.
